# Achilles tendon reconstruction with peroneus tendon transfer following epithelioid sarcoma resection: a rare case report at 5 years follow-up

**DOI:** 10.1186/s40634-020-00233-x

**Published:** 2020-03-24

**Authors:** Federica Mariotti, Silvio Caravelli, Massimiliano Mosca, Simone Massimi, Roberto Casadei, Stefano Zaffagnini

**Affiliations:** 1 IRCCS Istituto Ortopedico Rizzoli, Bologna, Italy; 2 Ospedale “Morgagni”, Forlì, Italy

## Abstract

**Background:**

Soft-tissue sarcomas (STS) are rare in hand and foot. In this paper we present a case of reconstruction of Achilles tendon defect with peroneus brevis transfer reinforced with medial gastrocnemius fascia and plantaris tendon after excision of a local recurrence of epithelioid sarcoma.

**Case presentation:**

Fifty-five years-old female. MRI showed a lump of 5 × 2,5 × 2 cm into Achille’s tendon with invasion of the anterior fat tissue but no invasion of the surrounding bones. The patient underwent excision of the tumour and reconstruction of the tendinous defect with peroneus brevis transfer. Surgical technique has been widely described.

**Discussion and conclusions:**

Epithelioid sarcoma arising from the Achilles tendon is an extremely rare malignant tumour in an atypical site and may easily be confused with other soft tissue masses. It presents a technical challenge because of the large tendon defect remaining following wide resection. Reconstruction with peroneus brevis transfer, reinforced with medial gastrocnemius fascia and plantaris tendon, restore appropriate structural continuity and resistance. Functional results are satisfactory.

## Introduction

Soft-tissue sarcomas (STS) are rare malignant mesenchymal tumours which arise in head, neck, retroperitoneum and limbs in most of the cases. They are rare in hand and foot [13]. The most frequent histotypes of STS in hand and foot are different from those observed in other locations: in the girdles and limbs liposarcoma and myxofibrosarcoma are the most common [17]. The most common STS reported in foot and hand are synovial sarcoma, epithelioid sarcoma (ES) and clear cell sarcoma [17]. Epithelioid sarcoma is a slow-growing lesion, more common in hand (58%) than foot (15%). It generally has a poor prognosis [6, 15, 16].

Generally, ES grows in subcutaneous layers, presenting as firm solitary or multinodular masses which may ulcerate and sometimes can be misdiagnosed as ulcerating squamous carcinoma. Deep-seated tumours are typically larger and firmly attached to tendons and fascia. Soft tissue sarcomas, arising from tendons and aponeuroses are rare and only a few cases are described in the literature [1, 8].

Because of its uncharacteristic clinical features, ES is easy to misdiagnose. Correct diagnosis is crucial not to income to inadequate excision [2, 7].

Soft tissue sarcomas of the foot represent a specific reconstructive challenge to the orthopaedic surgeon because of their proximity to critical structures such as tendons, ligaments, bones and neurovascular structure. In order to obtain wide margins, sacrifice of some of these structures is often necessary, with important functional impairment [12]. In addition, residual skin defect may require plastic surgeries.

In this paper we present a technical case of reconstruction of Achilles tendon defect with peroneus brevis transfer reinforced with medial gastrocnemius fascia and plantaris tendon after excision of a rare case of local recurrence of epithelioid sarcoma as an effective reconstructive strategy to obtain satisfactory functional results even after a wide resection as required.

## Case presentation

A 55 years-old female presented with insidious right foot pain and swelling over 6 months in absence of trauma. Symptoms worsened with walking and standing.

In April 2007 the patient underwent unplanned excision of the lump in the right Achilles’ tendon. Histology showed a myxofibrous inflammatory sarcoma grade 2 according to FNLCC classification.

Magnetic resonance imaging (MRI) was negative for local recurrence at 6 and 12 months after surgery. No further follow-up was performed during the next 4 years.

In August 2012 the patient was referred to our Institute for pain lasting 2 years. At the physical examination, the foot presented a lump, slightly painful, attached to the Achille’s tendon, with no signs of infection.

**Fig. 1:**
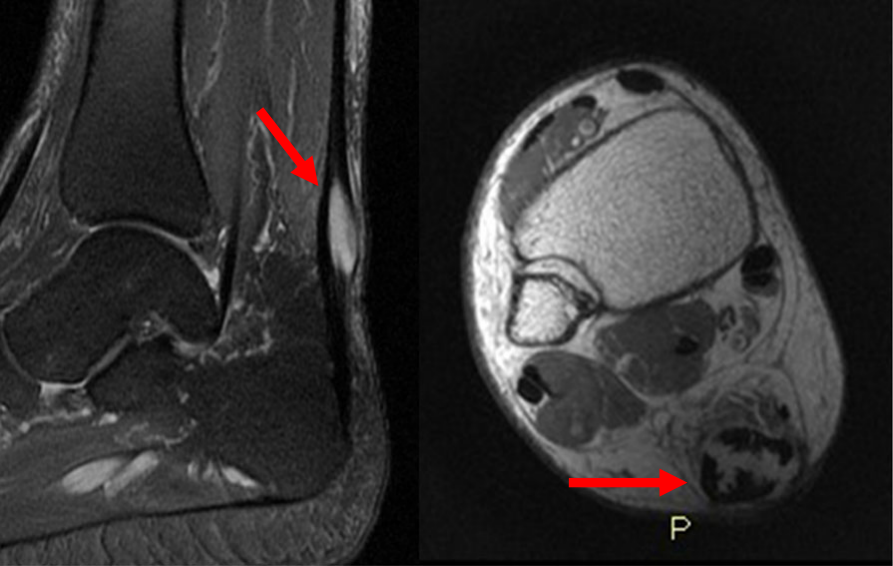
Pre-operative MRI showed a mass in the context of the middle third of Achilles tendon, with hyperintense signal in T2-weighted and T2-weighted fat-sat images

**Fig. 2:**
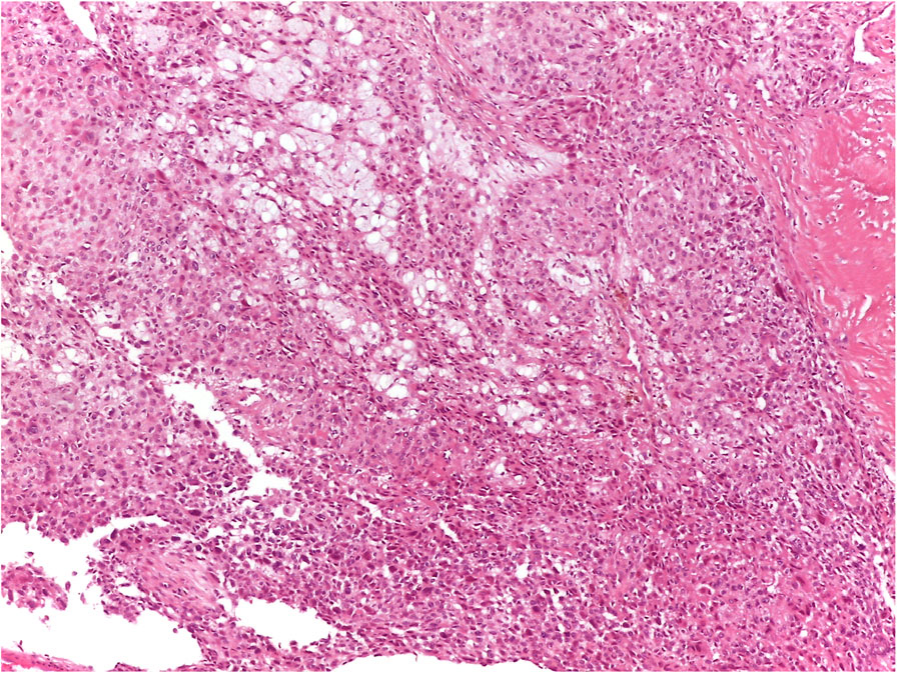
Malignant mesenchymal epithelioid cells growing in solid nests and trabeculae

**Fig. 3:**
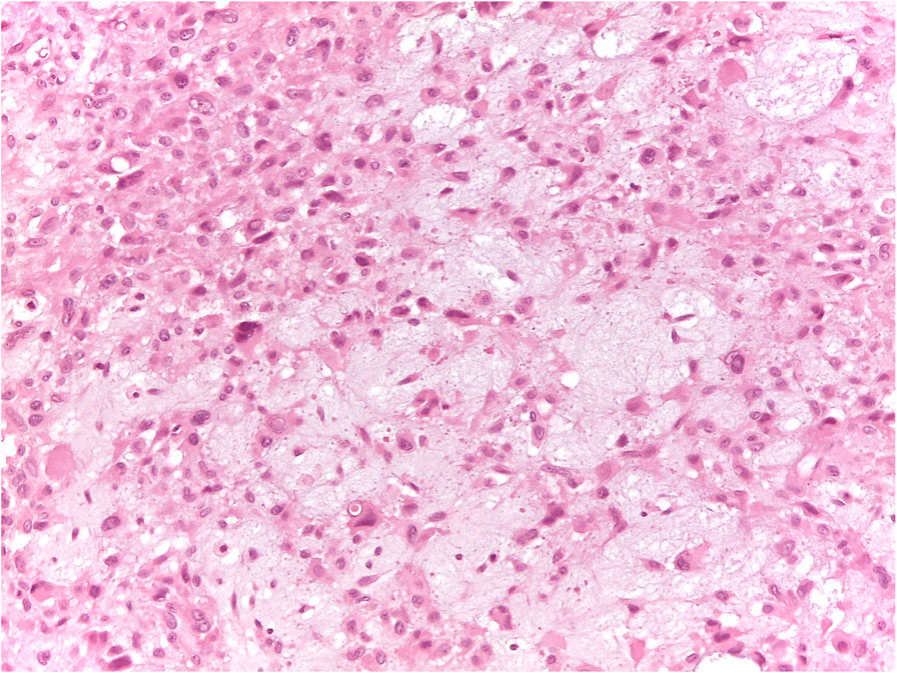
Tumoral cells showed enlarged, hyperchromatic atypical nuclei with evident pleomorphism. Intercelluler myxoid stroma was frequently present

In October 2014 MRI showed a lump of 5 × 2,5 × 2 cm into Achille’s tendon with invasion of the anterior fat tissue but no invasion of the surrounding bones (Fig. 1). A few days later, the patient was referred to our emergency ward for a distal tibia and bimalleolar fractures, which was treated through internal osteosynthesis. Before surgery, a needle biopsy was performed and histology showed a grade 2 epitheliomorphus sarcoma. After 1 months with no weight-bearing, x-rays showed initial fracture healing. Then the patient underwent tumor excision and reconstruction of the tendinous defect with peroneus brevis transfer. Histology showed epithelioid sarcoma, with immunohistochemical stains positive for epithelial membrane antigen (EMA), but INI 1 CD 31, S100, Mucine 4 and cytokeratin AE1/3 negative (Figs. 2, 3). Surgical margins were wide. Patient was discharged with a not walking plaster boot.

Forty days later the patient was re-admitted for plaster cast removal and to start adequate physiotherapic protocol. She was allowed to walk with progressive weight bearing, wearing ankle brace.

Follow up visits, Thoracic computerized tomography (CT) and foot and ankle MRI were then scheduled at every 4 months for 3 years, then every 6 months until the fifth year after surgery and then annually until the tenth year.

**Fig. 4:**
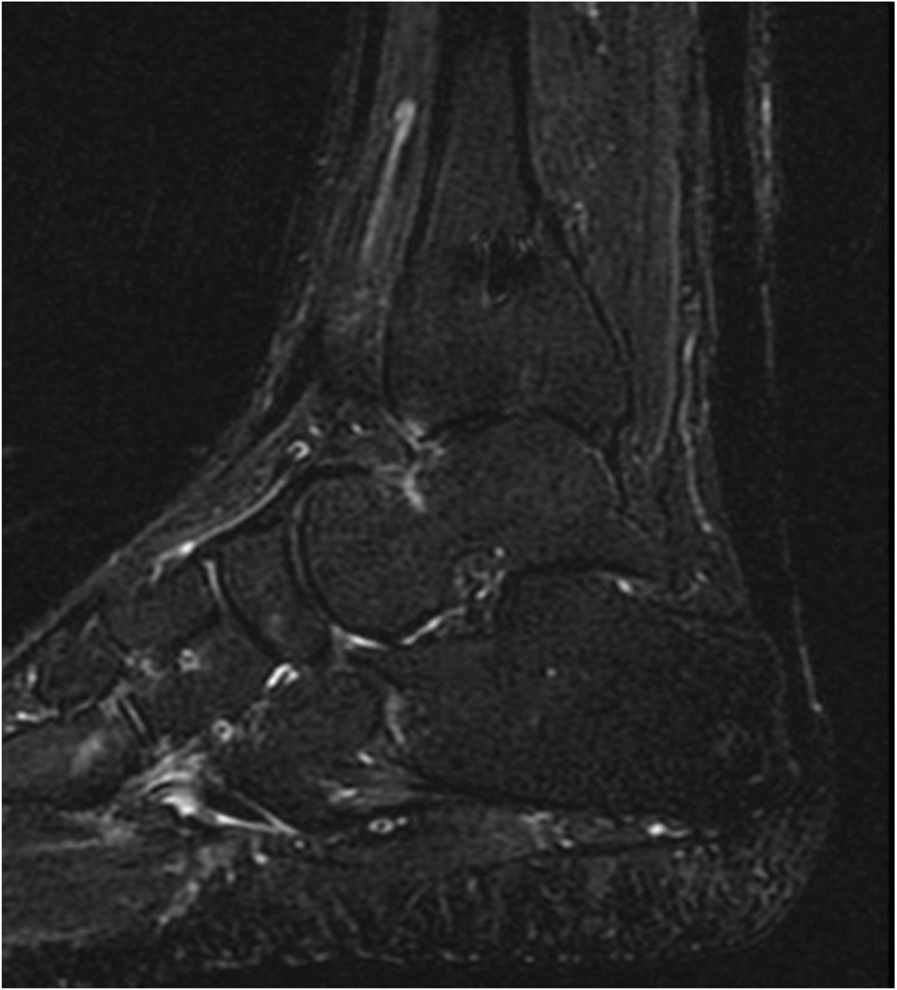
Post-operative MRI images on sagittal plane showed no recurrence and substantial continuity of the reconstruction

In January 2015 the patient presented a small area of skin defect and in February she was treated with negative pressure wound therapy for 3 months. In June the skin was completely healed, the MRI showed no sign of local recurrence (Fig. 4) and the CT scan was negative for metastasis.

One year later ankle range of movement was 45° in flexion, with a good ankle stability.

At the final follow-up at 5 years in 2019 there was no evidence of oncological disease and the patient was able to walk without pain nor limping, she could walk on tiptoe and remained a slight defect of 10° of flexion comparing to the contralateral side.

## Surgical technique

Pre-operatively, the insertion of the Achilles tendon on the calcaneum and the insertion of the peroneus brevis tendon over the base of the fifth metatarsal were identified as landmarks.

Under spinal anaesthesia, the patient was placed in prone position, with feet dangling from the end of the operating table and a thigh tourniquet was applied and inflated.

A 10 to 12 cm longitudinal skin incision was made directly on the median axis of the Achilles tendon; the previous incision was excised and lengthened for adequate exposure. Subcutaneous fat was penetrated by means of sharp dissection, sural nerve isolated and protected. The Achilles tendon was thus exposed, gently isolated along its length and visually examined. The tendon itself was adhered to surrounding paratenon and subcutaneous tissue and it appeared thickened and of reduced consistency. Ventrally, the loose tissues appeared undamaged and clean from gross traces of neoplasia, except for a small tumour expansion toward the anterior fat tissue.

We proceeded to separate the lesion from surrounding tissues and to resect en bloc a 6 cm segment of tendon, from the calcaneal apex to the proximal clean margin. Remaining tissue appeared healthy and skin edges viable.

Through the floor of the wound, the deep fascia overlying the compartment containing the peroneal muscles could be seen. The deep fascia overlying the peroneal tendons was incised and the peroneal tendons were mobilized. The distal portion of peroneus brevis muscle belly was exposed and its proximal end was identified.

**Fig. 5:**
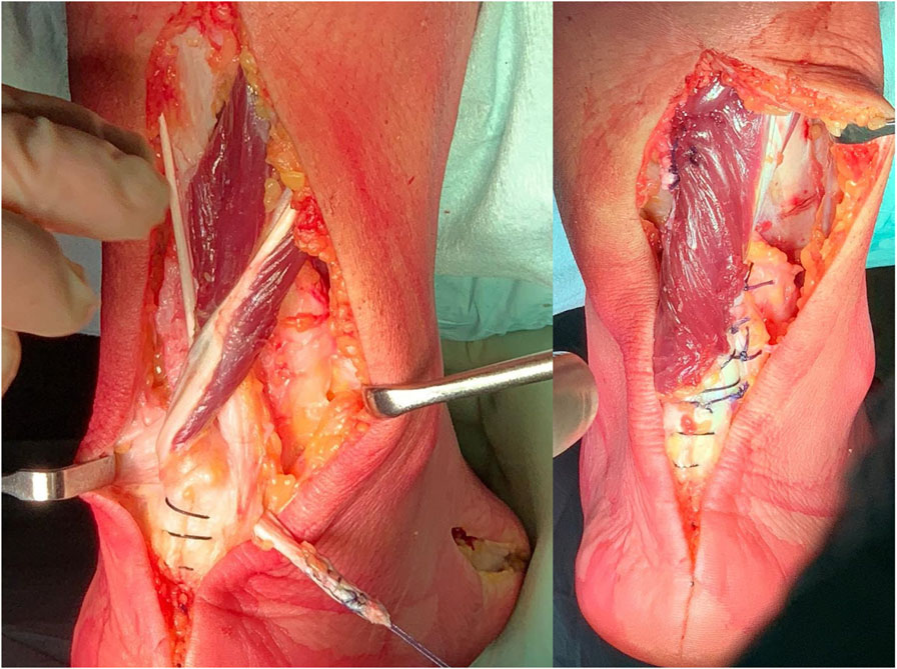
Surgical technique images. At left, peroneus brevis tendon crossing the calcaneal tunnel from medial to lateral (note the harvesting site on the lateral aspect of the base of the fifth metatarsal); at right, the final result of the reconstruction

A 2.5 cm longitudinal skin incision was made over the base of the fifth metatarsal and the tendon was identified and exposed. A stay suture was applied in the distal end of the tendon and this was detached from its insertion. After smooth dissection of the sheath, the tendon was delivered, by gentle traction, through the subcutaneous tissue, pulled through inferior retinaculum and basted in the distal end. The peroneus brevis tendon was then passed through a trans-osseous tunnel made through the posterior calcaneal tuberosity from medial-to-lateral and pulled proximally (Fig. 5). The distal end of peroneus brevis tendon was then secured medially, under tension, with absorbable n° 2 sutures to the proximal stump of the Achilles tendon to restore an appropriate structural continuity. Remaining length of the peroneus brevis was sutured at the lateral edge of the proximal stump.

To ensure better function and resistance, the medial part of the gastrocnemius fascia was detached and distally overturned to support the structure. Following, the plantaris tendon was harvested, weaved through the Achilles stump and sutured laterally to peroneus brevis tendon to augment the reconstruction. Tourniquet was deflated and hemostasis done. Subcutaneous tissue and the skin were closed, being careful of tension. The wound was dressed, a below-the-knee plaster cast applied with the foot in gravity equinus.

## Discussion and Findings

STS are uncommon, they represent only 1% of all adult malignancies and less than 10% are located in the distal limb [15, 18]. Epithelioid sarcoma was first described in 1970 by Enzinger [5], since then only few cases were documented in literature. It is a slow-growing high-grade sarcoma affecting young adults (20–40 years old) mostly. Upper limb location is dominant, while lower limbs represent only 32% of cases. 15% of lower limb ES occur in foot [13]. ES of tendons are extremely rare [6, 16]. Correct diagnosis and adequate treatment could be very challenging. Local recurrence rate is reported as frequent (63–92%) [15]^.^ Metastases from epithelioid sarcoma have been described in about 45% of patients with involvement of lungs and lymph nodes [3, 16]. The overall survival rates have been reported to be represented by 92.4%, 86.9% and 72.4% at 5, 10 and 15 years, respectively. Epithelioid sarcoma is still an only surgically curable disease [4].

The goals of malignant tumor resection in the foot and ankle are to obtain a wide surgical margin and to maintain a sensate, plantigrade and functional foot. Because of its rarity and its uncharacteristic clinical features, ES is easy to misdiagnose. Correct diagnosis is crucial not to income into inadequate excision and consequent need for revision surgery. According to Koulaxouzidis et al. [7] the so called “Whoops” procedure and consequent re-excision surgery are associated with more mutilating surgery and higher treatment costs. However how unplanned excision affects oncological outcomes is still controversial. According to Bianchi et al. [2] unplanned surgery does not compromise prognosis: they reported the same survival, local recurrence and distant metastases rate as STS primarily treated in a referral center, while according to Lewis et al. [9] residual tumor does influences local recurrence rate even after sufficient re-excision.

In Literature several surgical reconstruction techniques have been documented, more often used after a traumatic or chronic rupture. Maffulli et al. [10, 11] described peroneus brevis transfer technique for reconstruction of chronic tears of Achilles tendon. Those tears were up to a 6,5 cm tendon gap. In our case, the gap was about 6 cm. Of 16 patients treated, 1 patient had surgical wound infection healed by antibiotics therapy. All patients were able to walk with full weight bearing, walk on tiptoe, had good isometric plantar flexion strength and no-one had a re-rupture. Clinical outcome of strength and range of movement at final follow-up was comparable to the ones described by Maffulli [11]. We had a skin defect complication, healed by negative pressure wound therapy. Considering that the peroneal muscle provides 4% of the total plantar flexion and 28% of the hindfoot eversion capacity, the major concern about this technique are the reduced eversion and plantar flexion strength [10] however our functional outcome was satisfactory and the patient returned to pre-surgery daily activities. Sebastian et al. in 2007 compared mechanical properties of reconstructed Achilles tendon with Peroneus Brevis tendon or Flexor Hallucis Longus tendon, concluding that they perform similarly, regarding stiffness and energy, but Peroneus Brevis tendon transfer displayed a higher load to failure [14]. Furthermore, considering the large tendon defect in an oncological resection, the reinforce with medial gastrocnemius fascia and plantaris tendon we performed is useful to add strength to the reconstruction and minimize the risk of rupture.

## Conclusion

Epithelioid sarcoma arising from the Achilles tendon is an extremely rare malignant tumour in an atypical site and may easily be confused with other soft tissue masses. It presents a technical challenge because of the large tendon defect remaining following wide resection. Reconstruction with peroneus brevis transfer, reinforced with medial gastrocnemius fascia and plantaris tendon, restore appropriate structural continuity and resistance. Functional results are satisfactory.
